# Neuronal Representation of Ultraviolet Visual Stimuli in Mouse Primary Visual Cortex

**DOI:** 10.1038/srep12597

**Published:** 2015-07-29

**Authors:** Zhongchao Tan, Wenzhi Sun, Tsai-Wen Chen, Douglas Kim, Na Ji

**Affiliations:** 1 Janelia Research Campus, Howard Hughes Medical Institute, 19700 Helix Dr., Ashburn, Virginia, 20147

## Abstract

The mouse has become an important model for understanding the neural basis of visual perception. Although it has long been known that mouse lens transmits ultraviolet (UV) light and mouse opsins have absorption in the UV band, little is known about how UV visual information is processed in the mouse brain. Using a custom UV stimulation system and *in vivo* calcium imaging, we characterized the feature selectivity of layer 2/3 neurons in mouse primary visual cortex (V1). In adult mice, a comparable percentage of the neuronal population responds to UV and visible stimuli, with similar pattern selectivity and receptive field properties. In young mice, the orientation selectivity for UV stimuli increased steadily during development, but not direction selectivity. Our results suggest that, by expanding the spectral window through which the mouse can acquire visual information, UV sensitivity provides an important component for mouse vision.

UV vision is widespread in nature^1^,^2^ and used for a variety of essential tasks, such as navigation^3^, communication^4^, mate-selection^5^, and foraging^6^. Although in early vertebrates, UV vision was mediated by cone photoreceptors expressing UV-absorbing S-opsin (λ_max_ at 360 nm)^7^, many contemporary mammals lost their UV-sensitivity by shifting the peak absorption of their S-opsin into the visible spectrum. UV-absorbing S-opsin, however, is retained in the two largest mammalian orders, rodentia (e.g., mouse and rat) and chiroptera (e.g., the bats)^8^. In addition to S-opsin, the visible-absorbing rhodopsin and M-opsin also have substantial side absorption bands (“β-band”) in the UV^9^. In spite of the widespread UV sensitivity in the retina of many species^10^, the function of UV vision, especially in these mostly nocturnal animals, remains a mystery^11^,^12^, and little is known on how UV visual stimuli are processed in their central nervous systems.

The house mouse is an ideal model system to study the processing of UV stimuli in the brain. Behind lenses highly transparent in UV^13^, mouse retina is highly sensitive to UV light^14^. Like most mammals including diurnal species, the mouse retina is rod-dominated^15^. Although cones constitute only 3% of the photoreceptor population in mouse^16^, their density and absolute sensitivity are similar to those in the peripheral retina of primates^11^,^17^,^18^,^19^. S-opsin is expressed in two cone types: genuine S-cones, which express only S-opsin and synapse with S-cone-selective bipolar cells^20^, and co-expressing cones that also contain M-opsin^21^,^22^,^23^. Despite the coexpression of S- and M-opsins, mice can perform dichromatic color discrimination^24^, the most common form of mammalian color vision^25^. Another common feature of the mammalian retina^26^, spatially differential opsin expression, is also found in mice^21^,^22^,^23^,^27^,^28^,^29^,^30^,^31^: Within mouse retina, the expression level of S-opsin and M-opsin follows a dorsal-ventral gradient, with M-opsin dominant in the dorsal retina and S-opsin dominant in the ventral retina. Such a segregation of S- and M-opsin expression can support color opponency in retinal ganglion cells without requiring cone-type selective connectivity^32^,^33^,^34^.

The representation of visual stimuli in mouse central nervous system (CNS) has been extensively studied using visible stimuli^35^. Despite its low acuity, the mouse visual system is found to demonstrate many characteristics of cortical visual processing in higher mammals^18^,^36^. How UV stimuli are represented in the mouse CNS, however, is unknown, partly because typical visual stimulation methods do not deliver stimuli in UV. In this study, we constructed a UV projector to characterize UV-elicited responses of layer 2/3 neurons in mouse V1. Using *in vivo* two-photon calcium imaging^37^, we found that UV-evoked cortical responses were orientation-selective and exhibited similar spatiotemporal properties to those evoked by visible light. Half of all orientation-tuned neurons were exclusively selective to either UV or visible stimuli. The rest half were orientation-selective under both UV and visible stimulation. A small percentage of neurons were found to have chromatically opponent receptive fields. We also studied developmental trajectory of mouse UV vision and found that the percentage of orientation-selective neurons increased steadily during development.

## Results

### Cortical neurons show orientation selectivity to UV stimuli

**Figure 1:**
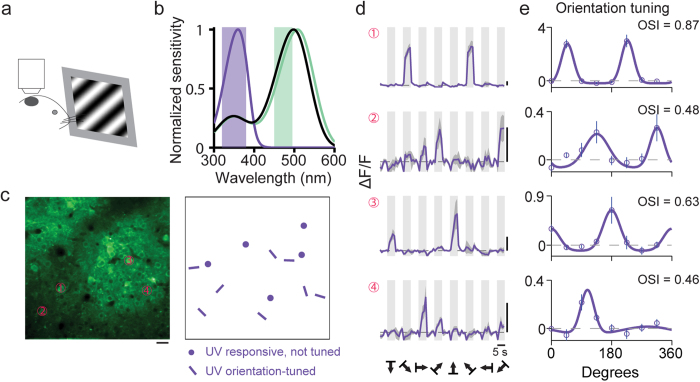
Neurons in mouse primary visual cortex respond to UV drifting gratings. (**a**) Two-photon fluorescence images were collected from GCaMP6-labeled neurons in primary visual cortex of mouse presented with drifting grating stimuli. (**b**) Normalized spectral sensitivity for S-opsin (purple), M-opsin (green), and rhodopsin (black). Shaded rectangles indicate the wavelength ranges of the UV and visible light sources. (**c**) Left, an example two-photon fluorescence image. Right, neuronal responses to UV drifting gratings over the same imaging field. Dots indicate neurons that exhibited significant responses but no orientation selectivity. Bars mark neurons that exhibited significant tuning for the oriented drifting gratings, with the bar orientation matching the orientation preference of each neuron. Scale bar: 20 μm. (**d**) Somatic calcium time courses for four example neurons labeled in (**c**). Light gray regions illustrate the 5-second duration of drifting grating stimulus. The average response across all 10 trials of a given stimulus condition is shown in purple trace, with the standard error across trials shaded in dark gray. Vertical scale bars: 40% ∆F/F. Horizontal scale bar: 5 s. (**e**) Orientation tuning curves for four neurons shown in **c** generated by averaging ∆F/F over the five-second window following the stimulus onset. Purple circles, mean; vertical purple line, error bar, SEM.; purple curve, fit to a double Gaussian function.

One important property of neurons in the mouse primary visual cortex is their selectivity to oriented visual stimuli, which has been well characterized using stimuli in the visible wavelength range. To determine whether L2/3 neurons are orientation selective to UV stimuli, we injected AAV-GCaMP6s virus into adult mouse primary visual cortex, and recorded cellular calcium responses to drifting sinusoidal gratings under UV illumination (100% contrast, spatial frequency 0.04 cyc/deg, temporal frequency 1 Hz) presented to the contralateral eye (Fig. 1a). Figure 1c shows an example imaging field, within which a subset of neurons were found to be responsive to UV gratings, with some of them showing orientation selectivity by having significantly different response amplitudes towards gratings of different orientations. Example somatic fluorescence time courses for four such neurons are shown in Fig. 1d. Orientation tuning curves were generated by plotting the response *R*_*i*_, the averaged ∆F/F over a five-second window following the onset of gratings drifting in direction *θ*_*i*_. The preferred orientation of each neuron was then identified by fitting its tuning curve with a double Gaussian function (Fig. 1e). Its orientation-selective index (OSI) was defined as 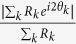. Among all UV-driven neurons, 69% (n = 174 out of 253 neurons, 7 animals) showed significant orientation selectivity (*P* < 0.05, one-way ANOVA test). We found little evidence for a clearly clustered organization of UV-sensitive cells with similar orientation selectivity, in line with the observations made in visible-responding neurons in rodent V1^38^.

### Cortical neurons responding to UV and visible light are interspersed in layer 2/3 and show similar orientation tuning properties

**Figure 2:**
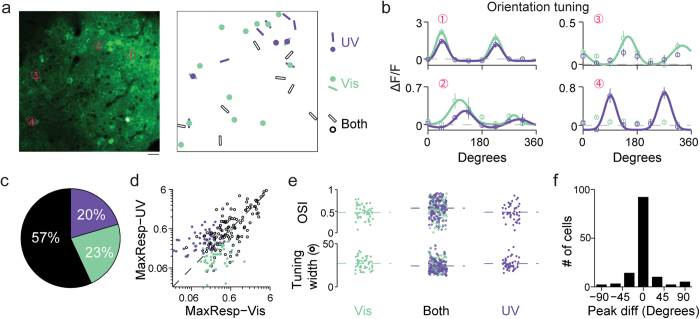
Subsets of neurons exhibit orientation selectivity for visible and UV light exclusively, while neurons selective to both have similar preferred orientation. (**a**) Left, two-photon image of neurons labeled with GCaMP6s. Scale bar: 20 μm. Right, responses to visible and UV drifting gratings. Dots, neurons with significant responses but no orientation selectivity to visual stimulation. Bars, neurons with significant orientation selectivity. Purple, UV-only responsive; green, visible-only responsive; black, responsive to both wavelengths. (**b**) Orientation tuning curves for four example neurons marked in **a**. Left, Two neurons orientation-tuned for both visible and UV wavelengths. Top right, a neuron exhibiting orientation selectivity to visible drifting grating exclusively. Bottom right, a neuron with orientation selectivity to UV drifting grating exclusively. Open circles, mean; vertical lines, error bar, SEM.; curves, double Gaussian fits to mean data. Green: visible; purple: UV. (**c**) Pie chart showing the percentage of neurons classified as orientation selective to only visible stimulation (green), only UV stimulation (purple), or both (black) (225 neurons in 7 animals). (**d**) Scatter plot of the maximal response to UV versus that to visible gratings for neurons orientation-selective to only visible (green dot), only UV (purple dot), and both visible-and-UV (open black circle) gratings. (**e**) Scatter plots of orientation selectivity index and tuning width. Dashed lines indicate mean values. (**f**) Histogram of difference in the preferred orientations of neurons orientation-selective to both UV and visible gratings.

We next investigate what relationship, if any, exists between the UV- and visible-responding neurons in layer 2/3 of mouse primary visual cortex. UV and visible drifting gratings were presented separately to the contralateral eye while evoked calcium signals were recorded at the center of the primary visual cortex (~2.7 mm lateral, 0.5 mm anterior to lambda). Within the imaging field of view of a few hundreds of microns, we found that, in terms of their spatial locations, the UV and visible responsive neurons are intermingled, with some responding to both UV and visible stimuli, while others responding to either UV or visible stimuli (Fig. 2a,b). We found that similar percentages of UV-responding and visible-responding neurons exhibited significant orientation selectivity (*P* < 0.05, one-way ANOVA test, 179 out of the 266 visible-responding neurons at 67% cf. 174 of the 253 UV-responding neurons at 69%). Out of 225 total orientation-tuned neurons, 57% (128) were tuned to both UV and visible stimuli (Fig. 2c). The remaining neurons were orientation-tuned in either UV (20%, 46 out of 225 neurons) or visible (23%, 51 out of 225 neurons) exclusively (Fig. 2b,c). For all these orientation-selective neurons, we plotted their maximal response to UV versus that to visible gratings (Fig. 2d). Across the population, neurons tuned to both wavelength ranges tended to distribute along the diagonal line while neurons tuned to single wavelength range were mainly dispersed on one side of the diagonal line.

**Figure 3:**
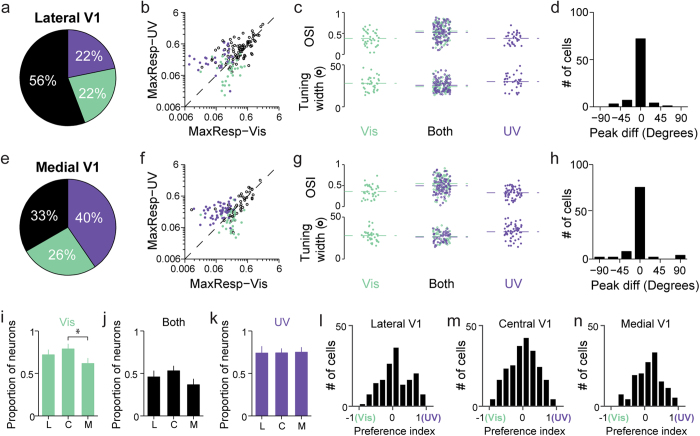
Neurons orientation-tuned to UV stimuli were distributed across areas of mouse V1 representing dorsal and ventral visual field. (**a**–**d**) Lateral V1 (dorsal visual field). (**a**) Pie chart showing the percentage of neurons classified as orientation selective to only visible stimulation (green), only UV stimulation (purple), or both (black) (156 neurons in 3 animals). (**b**) Scatter plot of the maximal response to UV versus that to visible gratings for neurons orientation-selective to only visible (green dot), only UV (purple dot), and both visible-and-UV (open black circle) gratings. (**c**) Scatter plots of orientation selectivity index and tuning width. Dashed lines indicate mean values. (**d**) Histogram of difference in the preferred orientations of neurons orientation-selective to both UV and visible gratings. (**e–h**) Medial V1 (ventral visual field) (141 neurons in 4 animals). (**i–k**) Proportions of neurons selective for visible, both and UV wavelength ranges among all orientation-selective neurons recorded in lateral (L), central (C) and medial (M) V1 (Mann-Whitney test, *P = 0.02). (**l–n**) Distributions of the response preference index for neurons located in lateral, central and medial V1, respectively.

We examined two indices reflecting tuning curve shapes: orientation selectivity index and tuning width (Fig. 2e). No differences were found in the distributions of orientation selectivity index and tuning width between exclusively visible- and UV-tuned neurons (Mann-Whitney test, *P* = 0.65, 0.92). For neurons tuned to both wavelengths, their mean orientation selectivity indices, calculated from their responses to either UV or visible stimuli, were significantly higher than the other two groups (Mann-Whitney test, *P* = 1 × 10^−3^, 4 × 10^−3^) and their mean tuning widths were significantly narrower (Mann-Whitney test, *P* = 6 × 10^−3^, 8 × 10^−3^). Majority of the UV-and-visibly-tuned neurons had similar preferred orientations, with differences in preferred orientation less than 45° (Fig. 2f). The same trends hold for neurons located more laterally (~3 mm lateral, 0.5 mm anterior to lambda; Fig. 3a–d) and medially (~2 mm lateral, 0.5 mm anterior to lambda; Fig. 3e–h), corresponding to the more dorsal and ventral regions of the visual field, respectively^39^. We did not observe significant response gradients towards either UV (Fig. 3k) or visible stimuli (Fig. 3i). In addition, a preference index, defined as (*R*_*UV*,max_−*R*_*Visible*,max_)/(*R*_*UV*,max_ + *R*_*Visible*,max_) with *R*_*UV*,max_ and *R*_*Visibles*,max_ being the peak UV and visible response, was calculated for neurons recorded at lateral, central and medial V1 (Fig. 3l–n). No significant difference was observed among distributions of the preference index across three regions of V1 (Kolmogorov-Smirnov test, *P* = 0.89, 0.21, 0.09). Therefore neurons located at the center of V1 were studied in the following experiments. Together, these results indicate that UV visual stimuli elicit substantial orientation-tuned responses from layer 2/3 neurons and suggest that, in mouse primary visual cortex, UV stimulation may be processed in a similar fashion to visible stimuli.

### UV and visible visual stimuli evoke neuronal responses with similar temporal and spatial frequency tuning properties

**Figure 4:**
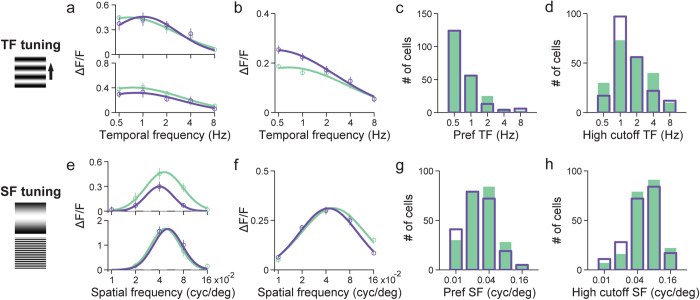
Temporal and spatial frequency tuning properties are similar for UV and visible stimuli. (**a**) Temporal frequency (TF) tuning curves for UV and visible upward drifting gratings for two example neurons. They were generated by averaging ∆F/F over the five second window following each stimulus onset. Open circles, mean; vertical lines, error bar, SEM; curves, log-Gaussian fits to mean data. Green: visible; purple: UV. (**b**) Average TF tuning curves over 211 and 218 neurons (N = 7 animals) that responded to UV and visible stimuli, respectively. (**c**) Distributions of preferred TF to UV (purple open bar) and visible stimuli (green solid bar). (**d**) Distributions of high cutoff TF to UV and visible stimuli. (**e**) Spatial frequency (SF) tuning curves for UV and visible upward drifting gratings are shown for two example neurons, generated by averaging ∆F/F over the five second window following each stimulus onset. (**f**) Average SF tuning curves over 247 and 226 neurons (N = 4 animals) that responded to UV and visible stimuli, respectively. (**g**) Distributions of preferred SF to UV and visible stimuli. (**h**) Distributions of high cutoff SF to UV and visible stimuli.

In addition to their similar orientation-selective responses, layer 2/3 cortical neurons also exhibited similar temporal and spatial tuning properties to UV and visible stimuli, as characterized by measuring their calcium responses to upward drifting gratings of varying temporal and spatial frequencies^40^,^41^. For temporal frequency responses, gratings at 5 temporal frequencies (0.5, 1, 2, 4, 8 Hz) and at the spatial frequency of 0.04 cyc/deg were used to evaluate 211 neurons activated by UV and 218 neurons activated by visible stimulation in 7 animals. Tuning curves were assessed for each neuron and then fitted with a log-Gaussian function to identify its preferred temporal frequency^41^. Figure 4a shows the temporal frequency tuning curves for two example neurons. For both UV and visible gratings, their responses peaked at low temporal frequencies and decreased significantly as temporal frequency increased. The same trend was observed in the average temporal frequency tuning curve pooled across the population of neurons (Fig. 4b). The preferred and high cutoff temporal frequency distributions for UV and visible stimulation were not significantly different (Fig. 4c,d) (*P* = 0.8, preferred; *P* = 0.4, high cutoff; Kolmogorov-Smirnov test).

For spatial frequency tuning, upward drifting gratings of 5 different spatial frequencies (0.01, 0.02, 0.04, 0.08, 0.16 cyc/deg) at the temporal frequency of 1 Hz were used to assess the spatial frequency tuning of 247 neurons activated by UV and 226 neurons activated by visible stimuli in 4 animals. Figure 4e shows the tuning profiles for two example neurons. Both of them had similar band-pass tuning profiles to UV and visible gratings, as did the average spatial frequency tuning curves pooled across neurons responding to either visible or UV stimulation (Fig. 4f). In terms of the preferred and the high cutoff spatial frequencies, their population distributions under UV and visible stimulation were not significantly different (Fig. 4g,h) (*P* = 0.2 preferred; *P* = 0.2, high cutoff; Kolmogorov-Smirnov test).

### Cortical neurons have overlapping and similarly sized receptive fields to UV and visible stimulation

**Figure 5:**
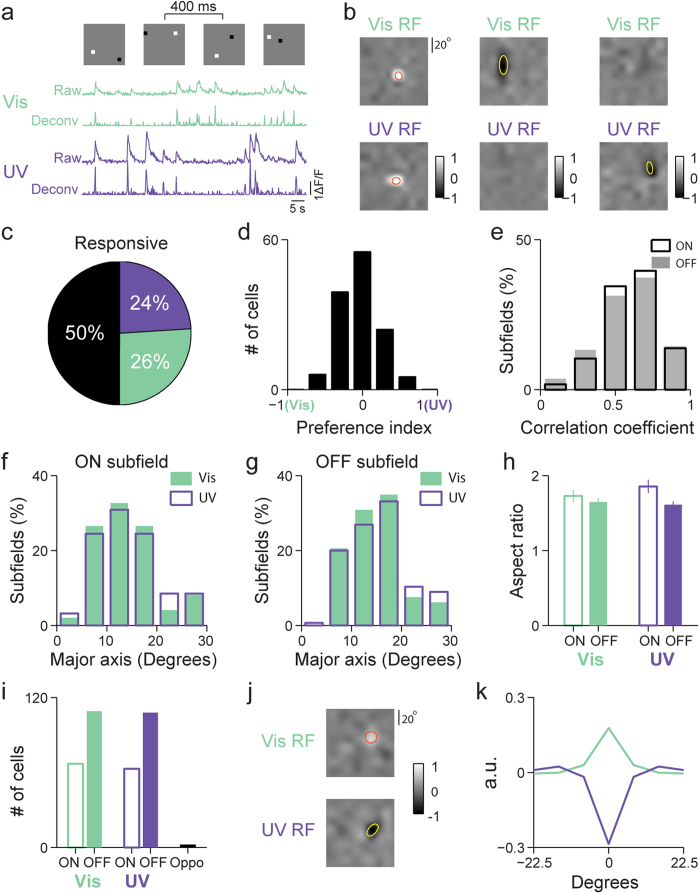
Neurons have overlapping and similarly-sized receptive fields to UV and visible stimulation. (**a**) Top, example sparse noise visual stimuli each lasting 400 ms. For one example neuron, raw calcium signal under visible (green) and UV (purple) stimulation (first and third traces) were plotted together with the deconvolved responses (second and forth traces). Vertical scale bar: 100% ∆F/F. Horizontal scale bar: 5 s. (**b**) Receptive fields (RF) for three example neurons, represented as the subtraction of their OFF subfields from their ON subfields, with one neuron responding to both wavelengths, one responding solely to visible, and one solely to UV, respectively. The gray scale was normalized to between −1 and 1 with 0 corresponding to balanced ON-/OFF-response. Top row: visible RFs; Bottom row: UV RFs. Yellow and red ellipses defined by the 2D Gaussian fitting parameters outline the RF. (**c**) Pie chart showing the percentage of neurons classified as responsive to only visible, only UV, or both wavelength ranges (258 neurons in 11 animals). (**d**) Histogram of the preference index (PI) for neurons responsive to both UV and visible stimuli. (**e**) Distributions of the correlation coefficients between visible and UV RFs for ON subfields (open box) and OFF subfields (shaded gray). (**f**) Width distributions of the major axis of ON subfields mapped using visible (green solid bar) and UV (purple open bar) stimuli. (**g**) Width distributions of the major axis of OFF subfields mapped using visible and UV stimuli. (**h**) Averages of the RF aspect ratios for ON and OFF subfields mapped using visible and UV stimuli. (**i**) Numbers of ON- and OFF-dominant neurons to visible (green) and UV (purple) stimuli, as well as chromatic opponent cells (black). (**j**) Receptive field of a VisibleON-UVOFF color-opponent neuron. Gray scale represents normalized response magnitude. The red and yellow line is 2D Gaussian fits of ON and OFF responses, respectively. (**k**) Radial spatial receptive field calculated as a function of distance from receptive field center for the neuron in **j**, with the overlaid visible and UV RFs exhibiting antagonistic spatial profiles.

The visual receptive field of a neuron is a two-dimensional (2D) region in visual space, within which a visual stimulus of appropriate structures can evoke neuronal activity^42^. Receptive fields of layer 2/3 neurons in mouse primary visual cortex were mapped using sparse-noise visual stimulation presented with UV and visible illumination, respectively. We injected AAV-GCaMP6f virus into the mouse primary visual cortex and monitored calcium transients of labeled layer 2/3 neurons during sparse-noise stimulation consisting of a pair of white (“ON” stimulation) and black (“OFF” stimulation) square pixels randomly distributed on a grid of gray background (Fig. 5a, upper panel). Neuronal responses were inferred from calcium transients using a fast nonnegative deconvolution method^43^ (Fig. 5a, lower panel). Jackknife analysis was used to resample the neuronal responses 10 times. Each time, spike-triggered averages for ON and OFF stimuli were calculated separately^44^ at a series of stimulus-response delays, and the ON or OFF receptive subfield yielding the highest peak response was chosen. The final receptive subfield, the average of the 10 ON or OFF subfields, was z-scored and included into further analysis if peak z-score for either ON or OFF subfield was above 5.

Of all the neurons whose RFs were obtained (n = 258, 11 animals), 26% responded only to visible sparse noise, 24% responded only to UV sparse noise, and 50% responded to both (Fig. 5c). Figure 5b shows the RFs for three example neurons, represented as the subtraction of their OFF subfields from their ON subfields, with one neuron responding to both wavelengths, one responding solely to visible, and one solely to UV, respectively. The preference indices for neurons responding to both wavelengths were calculated using the peak amplitudes in their UV and visible RFs, with most neurons having similar maximal responses to visible and UV stimuli with the peak of their distribution centered on zero (Fig. 5d). These neurons also have highly spatially overlapping and structurally correlated UV and visible RFs. Pearson correlation coefficients between the UV and visible RFs were calculated to quantify the extent of their spatial overlap. Across the population (Fig. 5e), the average Pearson’s correlation coefficients are 0.59 ± 0.03 for ON subfields and 0.56 ± 0.03 for OFF subfields (n = 129, mean ± SEM), respectively.

Fitting all the ON and OFF subfields each with a 2D Gaussian to quantify subfield size (i.e., the full widths for the major and minor axes) and shape (i.e., the aspect ratio defined as the ratio between the major and minor axis widths), we found that their distributions for UV and visible stimuli are statistically indistinguishable (Fig. 5f,g: *P* = 0.7, 0.9 for ON and OFF subfields, respectively, Kolmogorov-Smirnov test; Fig. 5h: *P* = 0.4, 0.9 for the aspect ratios of ON and OFF subfields, respectively, Kolmogorov-Smirnov test): for visible RFs, the population-averaged full widths of major and minor axes were 14.5° ± 7.1° and 9.3° ± 4.8° (n = 98, mean ± SEM) for ON subfields, 15.6° ± 7.1° and 10.5° ± 5.5° (n = 146) for OFF subfields, similar to RF sizes obtained in previous studies^36^,^45^,^46^. For UV RFs, the averaged full widths of major and minor axes were 14.9° ± 7.1° and 9.0° ± 5.0° (n = 94) for ON subfields, 16.6° ± 9.3° and 10.9° ± 6.0° (n = 145) for OFF subfields.

L2/3 neurons were classified into ON-dominant and OFF-dominant, by comparing the relative strengths of its responses to ON and OFF stimuli in their receptive fields. One-way ANOVAs were used to compare the maximal responses during the ON and OFF response time courses, and neurons were considered as significantly more sensitive to bright or dark stimuli when the *P* value was less than 0.05. For both UV and visible stimuli, there were consistently more OFF-dominant neurons than ON-dominant neurons (Fig. 5i), similar to previous observations in the primate visual cortex^47^. Interestingly, a small number of neurons were found to possess chromatically opponent responses^48^, which may underlie the dichromatic color vision observed in mouse^24^. Figure 5j shows the visible and UV RFs for a color-opponent neuron. Its radial receptive fields were calculated by averaging over concentric annuli^49^ and displayed as a function of the distance from receptive field center. As shown in Fig. 5k, this color-opponent neuron had antagonistic radial receptive field profile, behaving as OFF-dominant under UV illumination and ON-dominant under visible illumination. Of the 258 visually-responsive neurons whose RFs were measured, two neurons (~1%) were found to be significantly chromatic opponent, with their correlation coefficients between the visible and UV radial RFs being significantly negative (*P* < 0.05).

### UV-evoked visual response in young mice post eye opening

**Figure 6:**
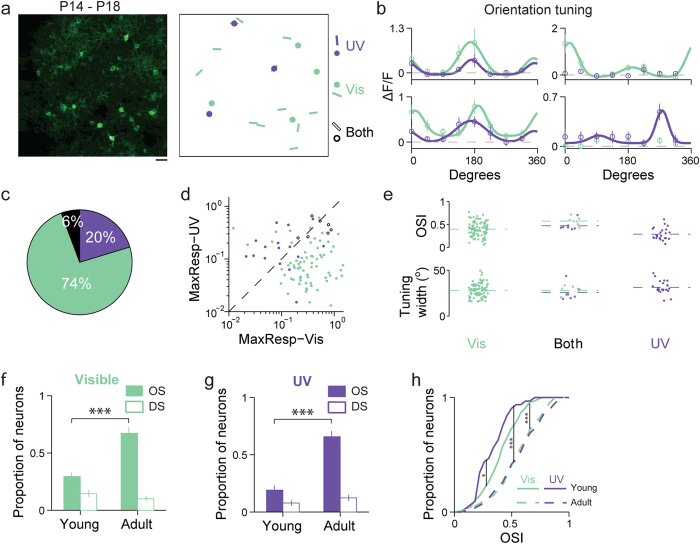
UV-evoked visual response in young mice post eye opening. (**a**) Left, two-photon image of neurons labeled with GCaMP6s. Scale bar: 20 μm. Right, responses to visible and UV drifting gratings. Dots, neurons with significant responses but no orientation selectivity. Bars, neurons with significant orientation selectivity. Purple, UV-only responsive; green, visible-only responsive; black, responsive to both wavelengths. (**b**) Orientation tuning curves for four example neurons. Left, neurons orientation-tuned to both visible and UV drifting gratings. Right, neurons exhibiting orientation selectivity exclusively to visible or UV drifting gratings, respectively. (**c**) Left, pie chart showing the percentage of neurons in young animals classified as orientation selective to only UV stimulation (purple), only visible stimulation (green), or both (black) (125 neurons in 12 young mice). (**d**) Scatter plot of the maximal response to UV versus that to visible gratings for neurons orientation-selective to only visible (green dot), only UV (purple dot), and both visible-and-UV (open black circle) gratings. (**e**) Scatter plots of orientation selectivity index and tuning width. Dashed lines indicate mean values. (**f**) Proportions of orientation-selective (OS, green solid bar) and of direction-selective (DS, green open bar) neurons among all responsive neurons recorded in young and adult animals under visible stimulation (Mann-Whitney test, ***P = 1 × 10^−6^). (**g**) Proportions of OS (purple solid bar) and of DS (purple open bar) neurons among all responsive neurons recorded in young and adult animals under UV stimulation (Mann-Whitney test, ***P = 1 × 10^−6^). (**h**) Cumulative distributions of orientation selectivity indices for UV (purple) and visible (green) in young (solid lines) and adult (dashed lines) mice (Kolmogorov-Smirnov test, *P = 0.04, ***P = 4 × 10^−8^, 4 × 10^−8^). Error bars in **f**–**g** indicate SEM across imaging sessions.

We also studied how L2/3 neurons in the primary visual cortex of young mice (P14-P18) responded to UV stimuli. L2/3 neurons were labeled with GCaMP6s via *in utero* viral injection, and their calcium activity evoked by full-field drifting gratings were recorded with two-photon fluorescence microscopy soon after eye opening at day P14-P18. Similar to the results obtained in adult mice, we found neurons that respond to either or both UV and visible stimuli (Fig. 6a,b). However, in young mice, a much smaller fraction of neurons exhibited orientation-tuned responses to UV gratings (Fig. 6c). We plotted the maximal response to UV versus that to visible gratings for all orientation-tuned neurons (Fig. 6d). Similar to that in the adult mice, across the population, neurons tuned to a single wavelength range were mainly dispersed on one side of the diagonal line, whereas neurons tuned in both wavelength ranges fall along the diagonal line. We also examined orientation selectivity index and tuning width (Fig. 6e). Exclusively UV-tuned neurons had significantly lower mean orientation selectivity index (Mann-Whitney test, *P* = 1 × 10^−3^, 3 × 10^−3^) than the other two groups. No significant difference was found in tuning width between exclusively UV- and visible-tuned neurons (Mann-Whitney test, *P* = 0.06).

We further investigated whether the UV-evoked cortical responses follow the same developmental trajectory in terms of their direction and orientation selectivity as their visible counterparts. In agreement with previous results^50^,^51^, with stimuli in the visible range, a much lower percentage of the visually-evoked neurons in young mice were found to possess significant orientation selectivity (one-way ANOVA test, *P* < 0.05) (29%, or 98 out of a total of 336 neurons, N = 12 young animals vs. 67% or 179 out of 266 neurons, N = 7 adult animals) (Fig. 6f). A similar trend was observed for UV stimuli, with the fraction of orientation-selective neurons increasing from 19% (32 out of 168 neurons) to 69% (174 out of 253 neurons) between young and adult mice (Fig. 6g). The increase of orientation selectivity during development is also reflected by the OSI cumulative distributions in young versus adult mice, with the young mice having, on average, significantly lower OSI than the adults under both UV and visible stimuli (Fig. 6h)^51^. The orientation selectivity in UV is also significantly lower than that in the visible (Fig. 6h). Many of the orientation-selective (OS) neurons in young mice are actually direction-selective (DS), a trend also observed with visible stimuli and explained previously by the differential development of orientation selectivity and direction selectivity in mice: at eye opening, most of the OS neurons are DS; strictly OS (but not DS) neurons appear during development with the proportion of DS neurons remaining unchanged^51^ (Fig. 6f,g,h). Our results suggest that UV-evoked visual processing follows similar developmental trajectory.

## Discussion

In mouse retina, UV response can be mediated by S-opsin through its peak absorption band as well as M-opsin and rhodopsin through their substantial β-band absorption in the UV range (Fig. 1b)^9^. The UV luminance level (173 R*/rod/s) used in our study does not saturate rod-mediated ganglion cell responses^29^. Therefore, it is possible that some of the cortical responses driven by UV stimuli may go through rod-mediated pathways. However, UV-evoked responses in cortex can be sustained by cones alone at our luminance level, as confirmed by two-photon calcium imaging experiment (Supplementary Fig. S1) in the primary visual cortex of Gnat^−/−^ mice that lack functional rods^52^. With only cones functional, neurons in Gnat^−/−^ mice show strong responses to UV stimuli under the same luminance condition and have similar orientation tuning properties to those observed in wild-type animals.

The β-band absorption of M-opsin and rhodopsin may underlie our inability to observe a UV-response gradient across V1 (Fig. 3). Under our illumination condition, in the dorsal retina, where M-opsin-dominated cones lie within a sea of rods, the visible stimuli act through the main absorption peak of M-cones whereas UV stimuli work through the β bands of both M-cones and rods; in the ventral retina, UV stimuli can evoke response through both S-opsin-dominated cones and rods, and there are still significant M-opsin present to mediate visible-evoked responses^29^,^31^. Therefore, under our illumination condition, both visible and UV stimuli can effectively stimulate the dorsal and ventral retina, as well as their retinotopic cortical areas.

Widespread coexpression of S and M-opsins was generally thought to be detrimental to color vision. Nevertheless, mice can make dichromatic color discrimination between UV and visible light^24^. Chromatic-opponent retinal ganglion cells have been discovered both *in vivo*^32^,^33^ and *ex vivo*^53^, and it has been proposed that the opsin distribution anisotropy in the form of opposing dorsoventral gradient in mouse retina is sufficient to generate chromatic opponency in RGCs without cone-type selective spatiotemporal processing^53^,^54^. It remains unclear whether and how these color discriminating RGCs contributes to observed dichromatic color vision in mice^53^, which requires studying chromatic processing in downstream neurons^48^. Using diffuse stimulation and single-unit recording, Ekesten and coworker found 1% of the neurons in mouse primary visual cortex to be chromatic-opponent^55^. Of all the visually responsive L2/3 neurons in our study, a similar percentage of them were found to have chromatic opponency as determined from their UV and visible receptive fields measured with sparse-noise stimuli. Each pixel in our sparse-noise stimulation extends a 7.5° visual angle, corresponding to a 0.25-mm stimulus diameter on retina itself, a size, according to recent work by Chang and coworkers, too small to evoke strong chromatic opponency in alpha-like RGCs^53^, which may explain the small percentage of chromatic opponent neurons found in our study. In addition to color opponency on single-neuron level, color perception may also be enabled through population coding via distributed activity across the cortical population of non-opponent neurons^56^,^57^. In our study, L2/3 neurons in V1 were found to possess a broad range of responses to visible and UV stimuli with substantial fractions classified as visible-only or UV-only neurons. Theoretically, their activity patterns may represent hues that can be decoded for color perception. A comprehensive investigation of color discrimination in mouse visual system is beyond the scope of this work. However, given the amenability of the mouse to many experimental techniques, we hope our results would motivate further study with mouse as a model system for dichromatic color signal processing.

In young mice immediately post eye-opening, a smaller percentage of orientation-selective neurons responded to our UV than visible stimuli. One possible explanation is that, in these mice, the postnatal development of cortical responses to UV is delayed compared to the response to visible, even though S-opsin immunoreactive cones are detected earlier than M-cones^58^. Interestingly, in human infants where S-opsin expression also precedes M/L-opsin^59^, the S-opsin mediated vision matures later compared to M/L-opsin vision^60^. However, several factors complicate this picture. At eye opening, the mouse lens still has areas of opacity and only becomes completely transparent one week later^61^. Not knowing the UV transmittance of these opaque lenses, we cannot be certain about the UV luminance level at retina. The stronger scattering for the shorter-wavelength UV light may also degrade the contrast of UV gratings on the retina more than visible gratings. Since luminance and contrast sensitivity of retinal ganglion cells in mouse retina after eye opening is lower than its adult counterpart^62^,^63^, the reduced cortical response to UV may be simply caused by less effective UV excitation. To elucidate the developmental timeline of UV vs. visible responses, one needs to characterize the ocular optical properties of young mice as well as compare light-evoked responses to UV and visible stimuli in *ex vivo* retina.

Our observations that neurons in mouse primary visual cortex respond to achromatic UV stimuli and have comparable spatiotemporal response properties as those to visible stimuli suggest that UV stimuli contribute to achromatic vision in similar ways to its visible counterparts. We found no significant difference in temporal frequency tuning of these L2/3 neurons under visible and UV stimuli. In both cases, the population-averaged TF tuning curves showed low-pass property, with the majority of the neurons exhibiting maximal responses at 0.5 Hz (the lowest TF presented), similar to previous observations in anaesthetized mice^39^,^64^. Because anesthesia suppresses responses at high temporal (and spatial) frequencies^41^,^65^, our results should only be interpreted in the context of UV and visible response comparison.

It is noteworthy that neurons in mouse primary visual cortex have similar SF responses and receptive field properties to UV and visible visual stimuli. It has been long proposed that S-cone vision is likely to have lower spatial resolution than vision mediated by longer wavelength cones, because the stronger scattering and aberration experienced by shorter wavelength light would degrade high-spatial-frequency information^2^,^66^. For example, there is a long-standing observation in human that blue vision mediated by S-cones is markedly inferior in acuity to that of the green and red vision, which renders humans essentially blue-blind for very small objects^67^,^68^,^69^. Neurons in the tree shrew primary visual cortex also have lower spatial frequency preferences in the blue S-cone responses than the response to longer wavelength stimuli^70^. The same trend has been speculated for mouse, whose S-opsin absorbs at even shorter wavelengths in ultraviolet^71^. However, the statistically identical spatial frequency tuning and receptive field properties for UV and visible stimuli suggest that UV-based vision in mouse has similar acuity to that based on visible light. This apparent contradiction becomes less surprising when we look at the S-opsin distribution in the photoreceptor mosaic: in human, S-cones are absent in the foveal center, which sustains the highest acuity vision and is protected from interference of short-wavelength irradiance by the blue- and UV-absorbing macular pigment, and are sparsely represented away from the fovea^72^; in tree shrew, the spacing between the S-cones is also much larger than the M cones^70^,^73^. For mouse, however, densities of the S-opsin-dominated cones and the M-opsin-dominated cones are comparable^27^, consistent with our results. With the already low visual acuity of mouse (~50–100 times worse than that of human)^74^,^75^, image quality degradation by mouse eye in the UV band may have negligible effects on its spatial acuity. However, because we only studied primary visual cortex, although our results suggest that S-cone mediated UV vision may sustain vision at an acuity as high as those based on longer wavelength cones, behavior experiments using UV stimuli would be needed to provide a definitive answer^74^.

Taken together, our results suggest that UV sensitivity provides an important component for mouse achromatic vision with similar feature selectivities to those for visible stimuli in primary visual cortex. Given their short life span and crepuscular/nocturnal lifestyle, retinal damage from UV irradiance would not be a serious concern, while UV vision capacity allows the expansion of the spectral window through which mouse can acquire visual information, especially during dusk and dawn when the relative intensity of UV light is highest^76^ and mice are most active^77^. The same principles may hold for other mammalian species with UV vision.

## Methods

### Animal Preparation

All procedures were in accordance with protocols approved by the Janelia Farm Research Campus Institutional Animal Care and Use Committee. Wild-type mice (C57BL/6Crl, Charles River) of either sex were used in all experiments. Adult animals were older than P60, and juvenile mice were between P14 and P18. During surgery, mice were anaesthetized with isoflurane-oxygen mixture (2% by volume in O_2_). A craniotomy was made over the left primary visual cortex (adult animal: center ~2.7 mm lateral, 0.5 mm anterior to lambda; young animal: center ~2.2 mm lateral, 0.4 mm anterior to lambda). The dura was left intact. For adult mice, 30 nL virus (AAV1-syn-GCaMP6s-WPRE-SV40 or AAV1-syn-GCaMP6f-WPRE-SV4) was slowly injected into the border between monocular and binocular regions at a depth of 200–250 μm below the pial surface to label L2/3 neurons for calcium imaging^78^. The injection system was comprised of a pulled glass pipette back-filled with mineral oil and connected to a one-axis oil hydraulic micromanipulator (Narishige). After injection, the cortex was covered with a double-layered glass coverslip, sealed in place with dental acrylic. The double-layered glass was comprised of a No. 2 glass coverslip (2 mm diameter) attached to a larger No. 1 glass coverslip (3.5 mm diameter) using UV-cured optical adhesives (Norland Optical Adhesives). A titanium head-post was attached to the skull with cyanoacrylate glue and dental acrylic to permit head fixation and two-photon imaging over the cranial window. Experiments were conducted 2–8 weeks after virus injections.

### In Utero Virus Injection

For juvenile mice, GCaMP6s was introduced into their cortex through *in utero* virus injection. E15-E16 timed-pregnant mice were deeply anesthetized with an isoflurane-oxygen mixture (2% volume in O_2_). The uterine horns were exposed. 345–690 nL virus (AAV1-syn-GCaMP6s-WPRE-SV40) with Fast Green (13–26 nl, 2.5 mg/ml) were injected through a pulled-glass capillary tube into the left embryonic cerebral ventricle in five consecutive pulses to label layer II/III neurons^79^.

### Visual Stimulation

Because equipment typically used for visual stimulation (e.g., computer monitors) do not emit in the UV range, visual stimuli were presented by back projection using a DLP^®^ projector on a screen made of UV-transmitting Teflon® film. The DLP^®^ projector was modified to run at 360 Hz by removing the color wheel. The lamp housing was replaced by a holder for a liquid light guide, and firmware modifications were made to ensure the equilength of all frames in the projected image (designed by Anthony Leonardo, Janelia Farm/HHMI, and Lightspeed Design Inc, model WXGA-360). Projected image luminance intensity varies linearly with the minimum being 2570 × smaller than the maximum. UV (320–380 nm, peak at 365 nm) and visible (450–495 nm, peak at 472 nm) light was generated by a UV lamp (Richard Wolf) and a LED light source (SugarCUBE), respectively, combined by a dichroic beamsplitter (FF409-Di03-25 × 36, Semrock), and delivered to the projector through the liquid light guide.

The screen was positioned 17 cm from the right eye, at ~40° to the long axis of the animal so that the receptive fields of the imaged neurons were at the center of the screen. The screen covered 75° × 75° degrees of visual space. For each protocol, UV and visible illuminations were used separately to generate stimulation patterns; therefore, all visual stimuli were single-colored in either UV or visible band. (In principle, heterochromatic UV-visible stimuli such as those used for cone-isolating stimulation^70^ can be generated by using two carefully-aligned projectors that project monochromatic UV and visible gratings onto the same screen. However, such stimulation is beyond the scope of this paper.) The power of the drifting grating stimuli measured using a UV-sensitive photodiode (S120VC, Tholabs, Ø9.5 mm) at the location of animal eyes was 437 nW/mm^2^ and 16.9 nW/mm^2^ for visible and UV light source, respectively. From the power, we then calculated the photoisomerization rates for rod, M-opsin-dominated cone, and S-opsin-dominated cone at the peak wavelengths of our UV and visible stimuli^19^:


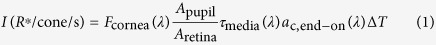


where *F*_cornea_(*λ*) is the flash strength of stimuli at wavelength *λ* specified in units of photons per mm^2^ at the cornea, *A*_pupil_ is 0.1 mm^2^ in mouse photopic vision^80^, *A*_retina_ is the retinal area occupied by the 75° target calculated as 4.9 mm^2^ 19, *τ*_media_(*λ*) is the transmission through ocular media, *a*_c,end-on_(*λ*) is the effective cross-sectional area of mouse cone for axially propagating light^19^,^22^, Δ*T* is 1 s in duration. Assuming that the transmission loss is dominated by the lens, *τ*_media_(*λ*) is 0.66 for UV (at 365 nm) and 0.89 for visible stimuli (at 472 nm)^81^. *a*_c,end-on_(*λ*)reflects the spectral sensitivity of the opsins and was calculated by^19^,^22^,^82^:


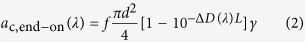


where *f* is a dimensionless factor that account for any light funneling by the inner segment, set at 1.24 for rods and 7 for cones; *d* is the outer segment diameter, set at 1.8 μm for rod and 1.5 μm for cones; *L* is the outer segment length, set at 25 μm for rods and 13 μm for cones; γ is quantum efficiency of photoisomerization, assumed to be the same for rods and cones and set at 0.66; Δ*D*(*λ*) is the axial optical density of opsins in the outer segment [o.d./μm] at wavelength λ. At *λ*_max_, Δ*D*(*λ*_max_) = 0.015 for rods and cones. Following the visual pigment templates proposed by Govardovskii *et al*.^9^, we have for rods, Δ*D*(365 nm) = 0.004 o.d./μm, Δ*D*(472 nm) = 0.013 o.d./μm; for M-opsin-dominated cones, Δ*D*(365 nm) = 0.004 o.d./μm, Δ*D*(472 nm) = 0.011 o.d./μm; for S-opsin-dominated cones, Δ*D*(365 nm) = 0.015 o.d./μm, Δ*D*(472 nm) = 0 o.d./μm. For rods, *a*_c,end-on_(365 nm) = 0.40 *μ*m^2^ and *a*_c,end-on_(472 nm) = 1.08 *μ*m^2^; for M-opsin-dominated cones, *a*_c,end-on_(365 nm) = 0.87 *μ*m^2^and *a*_c,end-on_(472 nm) = 2.31 *μ*m^2^; for S-opsin-dominated cones, *a*_c,end-on_(365 nm) = 2.91 *μ*m^2^and *a*_c,end-on_(472 nm) = 0 *μ*m^2^. Therefore, our UV and visible stimuli gave rise to *I*(R^*^/rod/s) of 173 at 365 nm and 2.0 × 10^4^ at 472 nm, *I*(R^*^/cone_M_/s) of 361 at 365 nm and 4.4 × 10^4^ at 472 nm, and *I*(R^*^/cone___S___/s) of 1.2 × 10^3^ at 365 nm and 0 at 472 nm, respectively. For measuring orientation tuning, full-field drifting sinusoidal gratings (100% contrast, 1 Hz, 0.04 cyc/deg) were presented in 8 directions^38^ in a pseudorandom sequence. Each stimulus was 5 s in duration with a 5 s gray-screen interstimulus interval. In the temporal frequency (TF) protocol, the stimulus set consisted of upward drifting gratings of five TFs (0.5, 1, 2, 4, 8 Hz) and at the SF of 0.04 cyc/deg. In the spatial frequency (SF) protocol, the stimulus set consisted of upward drifting gratings of five SFs (0.01, 0.02, 0.04, 0.08, 0.16 cyc/deg) and at the TF of 1 Hz^41^. A total of 10 blocks were presented in each measurement.

### Two-Photon Calcium Imaging

Mice were placed on a heating pad and kept anesthetized with 0.5% isoflurane-oxygen mixture and sedated with chlorprothixene (0.3 mg/ml, intramuscular injection, 40 μl and 20 μl for adult and young mice, respectively). UV-transparent silicone oil (polydimethylsiloxane) was applied to the surface of their eyes to prevent them from clouding. Imaging was performed with a custom-built two-photon microscope controlled by LabVIEW, as described previously^83^. A Ti:sapphire laser (Coherent) tuned to 900 nm was used for fluorescence excitation through a NA 0.8, 16× water dipping objective (Nikon). Imaging frames were acquired at ~7 Hz for receptive field mapping experiment^84^ and ~2 Hz for all the other experiments. The onset of each visual stimulus was marked by a small patch of bright pixels and detected by a photodetector, which then sent a voltage signal to trigger image acquisition.

### Tuning Curve Analysis

Image sequences were analyzed with custom programs written in MATLAB and LabVIEW. Image sequences obtained during 10 blocks of stimulation presentation were aligned to correct for motion-induced displacement in the XY plane. Somata of neurons were outlined by hand as regions of interest (ROIs). Neuropil contamination was corrected as *F*_cell___true_(*t*) = *F*_cell___measured_(*t*)−*r* × *F*_neuropil_(*t*), where *F*_cell___measured_(*t*) and *F*_cell___true_(*t*) are the neuronal signal before and after correction. The neuropil signal *F*_neuropil_(*t*) was measured by averaging the signal of all the pixels within a 20-μm region from the cell center but outside the somata outlines^85^. Scaling factor *r* was set at 0.7^78^. After averaging the *F*_cell___measured_(*t*) from the ten trials, the response *R*_*i*_ of each neuron was expressed as relative fluorescence changes (∆F/F) between the averages of the 2.5-second pre-stimulus baseline and the 5-second stimulation window from all pixels within specified ROIs. A neuron was considered as responsive if its activity during at least one visual stimulus was significantly higher than their activity during the interstimulus period by ANOVA test (*P* < 0.05)^86^. Under this criterion, 89% of L2/3 neurons in adult mice (total N = 299) respond to visible while 85% respond to UV stimuli. For young mice (N = 403), 84% respond to visible while 42% respond to UV stimuli.

Neurons were defined as orientation selective if there was significant response discrepancy across eight directions by ANOVA test (*P* < 0.05)^70^. For each neuron, statistical screening and parameter fitting were performed for UV and visible stimuli separately. For each neuron, its response *R*_*i*_, the averaged ∆F/F across five second stimulation window, was used to generate the tuning curve. To identify the preferred orientation of each neuron, the responses to drifting gratings were fitted with a 2-peak Gaussian function^86^,^87^:


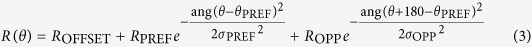


where *R*_OFFSET_ is a constant offset and *θ*_PREF_ is the preferred direction. *R*_PREF_ and *R*_OPP_ are the response amplitudes to the preferred and its opposite directions, respectively. *σ*_PREF_ and *σ*_OPP_ are the tuning widths for the preferred and its opposite directions, respectively. ang(*x*) = min(*x*, *x* −360, *x* + 360) wraps angular difference values onto the interval 0° to 180°. Orientation selectivity index (OSI) was calculated^88^ as 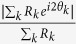 and reflected the spread of data over the polar coordinates. The index was close to 1 for exceptionally orientation-selective neuron and close to 0 for weakly selective neuron. Tuning width was defined as half-width at half-height at 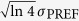. The direction selectivity index (DSI) was calculated as 
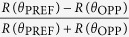
, where *R*(*θ*_PREF_) is the response in the preferred direction and *R*(*θ*_OPP_) is the response in the direction opposite to *θ*_PREF_. A neuron is classified as direction-selective if its DSI is larger than 0.5^51^.

Many neurons respond to both visible and UV stimuli. To quantify their relative responsiveness towards UV versus visible stimuli, we define a preference index as 
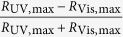
, where *R*_UV,max_ and *R*_Vis,max_ are the peak responses evoked by UV and visible stimuli, respectively. A preference index of 1(−1) indicates complete UV(Visible) dominance. Neurons giving identical responses to UV and visible stimuli have a preference index of 0.

In the SF and TF experiments, only responsive neurons were included for analysis, defined as those whose activity during one stimulation condition was significantly higher than that during the interstimulus period by ANOVA test (*P* < 0.05). To identify the preferred TF of each cell, responses were fitted by a log-gaussian function^41^,


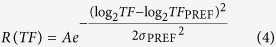


where *TF*_PREF_ is the preferred TF. The high cutoff TF is defined as the frequency where the fitted response declined to 3 dB of the peak response.

To identify the preferred SF of each cell, responses were also fitted by a log-gaussian function,


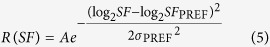


where *SF*_PREF_ is the preferred SF. The high cutoff SF is defined as the frequency where the fitted response declined to 3 dB of the peak response. For both the TF and SF experiments, only neurons with R-squared values superior to 0.8 were included.

### Receptive Field Mapping

RFs were obtained separately for UV and visible stimulations using *in vivo* two-photon calcium imaging^46^. GCaMP6f was used instead of GCaMP6s to utilize its faster response time^78^. Sparse-noise stimulation sequences were presented during two-photon calcium imaging. Each stimulus frame lasted for 400 ms and consisted of a pair of dark and bright square pixels randomly distributed within a gray 10 × 10 grid (in one case, 7 × 7 grid), extending ~75° of visual field. These frames were refreshed at 2.5 Hz and the total stimulation time was around 30 min for one wavelength range. Neuronal responses were inferred from calcium signals using the fast non-negative deconvolution method^43^. Jackknife analysis was then used to resample the neuronal responses 10 times and each time omitting a different 10% of the response^45^,^89^. For each resampled response set, spike-triggered averages for ON and OFF stimuli were calculated separately^44^ at various time delays from −2 to 5 frames prior to neuronal responses. The ON and OFF receptive subfields obtained at the time delays that yielded the highest peak response amplitudes were chosen. The final receptive subfields were the averages of these 10 pairs of receptive subfields. Only subfields that pass thresholding at 5 z-scores were included for further analysis. The final receptive field was calculated by subtracting the OFF subfield from the ON subfield.

In order to compare the similarity of RF structures between visible and UV stimulation, Pearson correlation coefficient (*r*) was calculated between visible and UV RF’s for their ON and OFF subfields, respectively^90^:


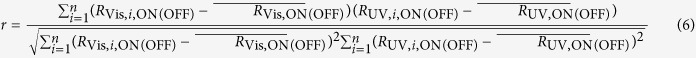


where *R*_Vis(UV),*i*,ON(OFF)_ is the visible(UV) response at each grid location *i* of the ON(OFF) receptive subfield^90^.

For easier visualization, RFs were smoothed with cubic spline interpolation while all quantitative analyses were performed on uninterpolated RFs^46^. The ON and OFF subfields were each independently fitted with a 2D Gaussian function^90^ only if their peak z-scores were above five:


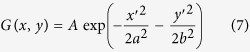


where *x*′ and *y*′ are the 2D rotational transformations of space coordinates *x* and *y* at angle *θ*, along which the Gaussian function is oriented. Consequently, RF and their subfields were depicted by the area enclosed by the ellipse with *a* and *b* as its semimajor and semiminor axes. The aspect ratio of the receptive subfield was defined as the ratio between *a* and *b*.

The jackknife analysis above allowed us to compare the response magnitudes to ON and OFF stimuli, in order to determine whether a responsive neuron is ON-dominant, OFF-dominant, or equally responsive to ON and OFF stimuli. One-way ANOVA test was performed for the maximal responses at the optimal stimulus-response delay during the ON and OFF time courses. The difference between ON and OFF responses was considered significant if the statistical test gave a *P*-value less than 0.05.

For neurons that have RFs measured in both UV and visible, a radial spatial receptive field was calculated by averaging across concentric annuli, as a function of the distance from the center of its receptive field. The correlation coefficient between the visible and UV radial receptive fields was used to determine whether spatially antagonistic color-opponent characteristics exist between visible and UV receptive fields. A neuron was considered color-opponent if it had a significantly (*P* < 0.05) negative correlation coefficient.

## Additional Information

**How to cite this article**: Tan, Z. *et al.* Neuronal Representation of Ultraviolet Visual Stimuli in Mouse Primary Visual Cortex. *Sci. Rep.* **5**, 12597; doi: 10.1038/srep12597 (2015).

## Supplementary Material

Supplementary Information

## References

[b1] Jacobs G. H. Ultraviolet vision in vertebrates. Amer. Zool. 32, 544–554 (1992).

[b2] Tovee M. J. Ultra-violet photoreceptors in the animal kingdom: their distribution and function. Trends Ecol. Evol. 10, 455–460 (1995). 21237101 10.1016/s0169-5347(00)89179-x

[b3] Menzel R. & Snyder A. Polarised light detection in the bee, Apis mellifera. J. Comp. Physiol. 88, 247–270 (1974).

[b4] Fleishman L. J., Loew E. R. & Leal M. Ultraviolet vision in lizards. Nature 365, 397–397 (1993).

[b5] Bennett A. T. D., Cuthill I. C., Partridge J. C. & Maier E. J. Ultraviolet vision and mate choice in zebra finches. Nature 380, 433–435 (1996).

[b6] Viitala J., Korplmaki E., Palokangas P. & Koivula M. Attraction of kestrels to vole scent marks visible in ultraviolet light. Nature 373, 425–427 (1995).

[b7] Yokoyama S. Evolution of dim-light and color vision pigments. Annu. Rev. Genomics Hum. Genet. 9, 259–282 (2008). 18544031 10.1146/annurev.genom.9.081307.164228

[b8] Hunt D. M. & Peichl L. S cones: evolution, retinal distribution, development, and spectral sensitivity. Vis. Neurosci. 31, 115–138 (2014). 23895771 10.1017/S0952523813000242

[b9] Govardovskii V. I., Fyhrquist N., Reuter T., Kuzmin D. G. & Donner K. In search of the visual pigment template. Vis. Neurosci. 17, 509–528 (2000). 11016572 10.1017/s0952523800174036

[b10] Douglas R. H. & Jeffery G. The spectral transmission of ocular media suggests ultraviolet sensitivity is widespread among mammals. Proc. Roy. Soc. B 281, 20132995 (2014). 10.1098/rspb.2013.2995 PMC4027392 24552839

[b11] Gouras P. & Ekesten B. Why do mice have ultra-violet vision? Exp. Eye. Res. 79, 887–892 (2004). 15642326 10.1016/j.exer.2004.06.031

[b12] Jacobs G. H. Losses of functional opsin genes, short-wavelength cone photopigments, and color vision—A significant trend in the evolution of mammalian vision. Vis. Neurosci. 30, 39–53 (2013). 23286388 10.1017/S0952523812000429

[b13] Lei B. & Yao G. Spectral attenuation of the mouse, rat, pig and human lenses from wavelengths 360 nm to 1020 nm. Exp. Eye Res. 83, 610–614 (2006). 16682025 10.1016/j.exer.2006.02.013

[b14] Jacobs G. H., Neitz J. & Deegan J. F. 2nd Retinal receptors in rodents maximally sensitive to ultraviolet light. Nature 353, 655–656 (1991). 1922382 10.1038/353655a0

[b15] Peichl L. Diversity of mammalian photoreceptor properties: Adaptations to habitat and lifestyle? Anat. Rec. A Discov. Mol. Cell Evol. Biol. 287A, 1001–1012 (2005). 16200646 10.1002/ar.a.20262

[b16] Carter-Dawson L. D. & Lavail M. M. Rods and cones in the mouse retina. I. Structural analysis using light and electron microscopy. J. Comp. Neurol. 188, 245–262 (1979). 500858 10.1002/cne.901880204

[b17] Jeon C.-J., Strettoi E. & Masland R. H. The major cell populations of the mouse retina. J. Neurosci. 18, 8936–8946 (1998). 9786999 10.1523/JNEUROSCI.18-21-08936.1998 PMC6793518

[b18] Umino Y., Solessio E. & Barlow R. B. Speed, spatial, and temporal tuning of rod and cone vision in mouse. J. Neurosci. 28, 189–198 (2008). 18171936 10.1523/JNEUROSCI.3551-07.2008 PMC2847259

[b19] Naarendorp F. *et al.* Dark light, rod saturation, and the absolute and incremental sensitivity of mouse cone vision. J. Neurosci. 30, 12495–12507 (2010). 20844144 10.1523/JNEUROSCI.2186-10.2010 PMC3423338

[b20] Haverkamp S. *et al.* The primordial, blue-cone color system of the mouse retina. J. Neurosci. 25, 5438–5445 (2005). 15930394 10.1523/JNEUROSCI.1117-05.2005 PMC6725002

[b21] Röhlich P., van Veen T. & Szél Á. Two different visual pigments in one retinal cone cell. Neuron 13, 1159–1166 (1994). 7946352 10.1016/0896-6273(94)90053-1

[b22] Lyubarsky A. L., Falsini B., Pennesi M. E., Valentini P. & Pugh E. N. Jr. UV- and midwave-sensitive cone-driven retinal responses of the mouse: a possible phenotype for coexpression of cone photopigments. J. Neurosci. 19, 442–455 (1999). 9870972 10.1523/JNEUROSCI.19-01-00442.1999 PMC6782392

[b23] Applebury M. L. *et al.* The murine cone photoreceptor: a single cone type expresses both S and M opsins with retinal spatial patterning. Neuron 27, 513–523 (2000). 11055434 10.1016/s0896-6273(00)00062-3

[b24] Jacobs G. H., Williams G. A. & Fenwick J. A. Influence of cone pigment coexpression on spectral sensitivity and color vision in the mouse. Vision Res. 44, 1615–1622 (2004). 15135998 10.1016/j.visres.2004.01.016

[b25] Jacobs G. H. The distribution and nature of colour vision among the mammals. Biol. Rev. 68, 413–471 (1993). 8347768 10.1111/j.1469-185x.1993.tb00738.x

[b26] Temple S. E. Why different regions of the retina have different spectral sensitivities: a review of mechanisms and functional significance of intraretinal variability in spectral sensitivity in vertebrates. Vis. Neurosci. 28, 281–293 (2011). 21838935 10.1017/S0952523811000113

[b27] Szél Á. *et al.* Unique topographic separation of two spectral classes of cones in the mouse retina. J. Comp. Neurol. 325, 327–342 (1992). 1447405 10.1002/cne.903250302

[b28] Calderone J. B. & Jacobs G. H. Regional variations in the relative sensitivity to UV light in the mouse retina. Vis. Neurosci. 12, 463–468 (1995). 7654604 10.1017/s0952523800008361

[b29] Wang Y. V., Weick M. & Demb J. B. Spectral and temporal sensitivity of cone-mediated responses in mouse retinal ganglion cells. J. Neurosci. 31, 7670–7681 (2011). 21613480 10.1523/JNEUROSCI.0629-11.2011 PMC3122925

[b30] Liu B. H. *et al.* Intervening inhibition underlies simple-cell receptive field structure in visual cortex. Nat. Neurosci. 13, 89–96 (2010). 19946318 10.1038/nn.2443 PMC2818750

[b31] Baden T. *et al.* A tale of two retinal domains: near-optimal sampling of achromatic contrasts in natural scenes through asymmetric photoreceptor distribution. Neuron 80, 1206–1217 (2013). 24314730 10.1016/j.neuron.2013.09.030

[b32] Ekesten B., Gouras P. & Yamamoto S. Cone inputs to murine retinal ganglion cells. Vis. Res 40, 2573–2577 (2000). 10958909 10.1016/s0042-6989(00)00122-x

[b33] Ekesten B. & Gouras P. Cone and rod inputs to murine retinal ganglion cells: evidence of cone opsin specific channels. Vis. Neurosci. 22, 893–903 (2005). 16469196 10.1017/S0952523805226172

[b34] Chang L., Breuninger T. & Euler T. Chromatic coding from cone-type unselective circuits in the mouse retina. Neuron 77, 559–571 (2013). 23395380 10.1016/j.neuron.2012.12.012

[b35] Huberman A. D. & Niell C. M. What can mice tell us about how vision works? Trends Neurosci. 34, 464–473 (2011). 21840069 10.1016/j.tins.2011.07.002 PMC3371366

[b36] Niell C. M. & Stryker M. P. Highly selective receptive fields in mouse visual cortex. J. Neurosci. 28, 7520–7536 (2008). 18650330 10.1523/JNEUROSCI.0623-08.2008 PMC3040721

[b37] Stosiek C., Garaschuk O., Holthoff K. & Konnerth A. *In vivo* two-photon calcium imaging of neuronal networks. Proc. Natl. Acad. Sci. USA. 100, 7319–7324 (2003). 12777621 10.1073/pnas.1232232100 PMC165873

[b38] Ohki K., Chung S., Ch’ng Y. H., Kara P. & Reid R. C. Functional imaging with cellular resolution reveals precise micro-architecture in visual cortex. Nature 433, 597–603 (2005). 15660108 10.1038/nature03274

[b39] Marshel J. H., Garrett M. E., Nauhaus I. & Callaway E. M. Functional specialization of seven mouse visual cortical areas. Neuron 72, 1040–1054 (2011). 22196338 10.1016/j.neuron.2011.12.004 PMC3248795

[b40] Glickfeld L. L., Andermann M. L., Bonin V. & Reid R. C. Cortico-cortical projections in mouse visual cortex are functionally target specific. Nat. Neurosci. 16, 219–226 (2013). 23292681 10.1038/nn.3300 PMC3808876

[b41] Andermann M. L., Kerlin A. M., Roumis D. K., Glickfeld L. L. & Reid R. C. Functional specialization of mouse higher visual cortical areas. Neuron 72, 1025–1039 (2011). 22196337 10.1016/j.neuron.2011.11.013 PMC3876958

[b42] Hubel D. H. & Wiesel T. N. Receptive fields of single neurones in the cat’s striate cortex. J. Physiol. 148, 574–591 (1959). 14403679 10.1113/jphysiol.1959.sp006308 PMC1363130

[b43] Vogelstein J. T. *et al.* Fast nonnegative deconvolution for spike train inference from population calcium imaging. J. Neurophysiol. 104, 3691–3704 (2010). 20554834 10.1152/jn.01073.2009 PMC3007657

[b44] Reid R. C. & Shapley R. M. Spatial structure of cone inputs to receptive fields in primate lateral geniculate nucleus. Nature 356, 716–718 (1992). 1570016 10.1038/356716a0

[b45] Bonin V., Histed M. H., Yurgenson S. & Reid R. C. Local diversity and fine-scale organization of receptive fields in mouse visual cortex. J. Neurosci. 31, 18506–18521 (2011). 22171051 10.1523/JNEUROSCI.2974-11.2011 PMC3758577

[b46] Smith S. L. & Hausser M. Parallel processing of visual space by neighboring neurons in mouse visual cortex. Nat. Neurosci. 13, 1144–1149 (2010). 20711183 10.1038/nn.2620 PMC2999824

[b47] Yeh C. I., Xing D. & Shapley R. M. “Black” responses dominate macaque primary visual cortex v1. J. Neurosci. 29, 11753–11760 (2009). 19776262 10.1523/JNEUROSCI.1991-09.2009 PMC2796834

[b48] Shapley R. & Hawken M. J. Color in the cortex: single- and double-opponent cells. Vision Res. 51, 701–717 (2011). 21333672 10.1016/j.visres.2011.02.012 PMC3121536

[b49] Reid R. C. & Shapley R. M. Space and time maps of cone photoreceptor signals in macaque lateral geniculate nucleus. J. Neurosci. 22, 6158–6175 (2002). 12122075 10.1523/JNEUROSCI.22-14-06158.2002 PMC6757940

[b50] Fagiolini M., Pizzorusso T., Berardi N., Domenici L. & Maffei L. Functional postnatal development of the rat primary visual cortex and the role of visual experience: dark rearing and monocular deprivation. Vision Res. 34, 709–720 (1994). 8160387 10.1016/0042-6989(94)90210-0

[b51] Rochefort N. L. *et al.* Development of direction selectivity in mouse cortical neurons. Neuron 71, 425–432 (2011). 21835340 10.1016/j.neuron.2011.06.013

[b52] Calvert P. D. *et al.* Phototransduction in transgenic mice after targeted deletion of the rod transducin α-subunit. Proc. Natl. Acad. Sci. USA 97, 13913–13918 (2000). 11095744 10.1073/pnas.250478897 PMC17675

[b53] Chang L., Breuninger T. & Euler T. Chromatic coding from cone-type unselective circuits in the mouse retina. Neuron 77, 559–571 (2013). 23395380 10.1016/j.neuron.2012.12.012

[b54] Neitz J. & Neitz M. The genetics of normal and defective color vision. Vision Res. 51, 633–651 (2011). 21167193 10.1016/j.visres.2010.12.002 PMC3075382

[b55] Ekesten B. & Gouras P. Cone inputs to murine striate cortex. BMC neurosci. 9, 113 (2008). 19014590 10.1186/1471-2202-9-113 PMC2615778

[b56] Wachtler T., Sejnowski T. J. & Albright T. D. Representation of color stimuli in awake macaque primary visual cortex. Neuron 37, 681–691 (2003). 12597864 10.1016/s0896-6273(03)00035-7 PMC2948212

[b57] Lehky S. R. & Sejnowski T. J. Seeing white: Qualia in the context of decoding population codes. Neural Comput. 11, 1261–1280 (1999). 10423495 10.1162/089976699300016232

[b58] Szel A., Rohlich P., Mieziewska K., Aguirre G. & van Veen T. Spatial and temporal differences between the expression of short- and middle-wave sensitive cone pigments in the mouse retina: a developmental study. J. Comp. Neurol. 331, 564–577 (1993). 8509512 10.1002/cne.903310411

[b59] Swaroop A., Kim D. & Forrest D. Transcriptional regulation of photoreceptor development and homeostasis in the mammalian retina. Nat. Rev. Neurosci. 11, 563–576 (2010). 20648062 10.1038/nrn2880 PMC11346175

[b60] Kellman P. J. & Arterberry M. E. in Handbook of child psychology 6^th^ edn, Vol. 2 (eds Kuhn D. *et al.* ) Ch. 3, 109–160 (John Wiley & Sons, Inc., 2007).

[b61] Son A. I. *et al.* Further analysis of the lens of ephrin-A5-/- mice: development of postnatal defects. Mol. Vis. 19, 254–266 (2013). 23401654 PMC3566898

[b62] Koehler C. L., Akimov N. P. & Renteria R. C. Receptive field center size decreases and firing properties mature in ON and OFF retinal ganglion cells after eye opening in the mouse. J. Neurophysiol. 106, 895–904 (2011). 21613583 10.1152/jn.01046.2010 PMC3154829

[b63] Akimov N. P. & Renteria R. C. Dark rearing alters the normal development of spatiotemporal response properties but not of contrast detection threshold in mouse retinal ganglion cells. Dev. Neurobiol. 74, 692–706 (2014). 24408883 10.1002/dneu.22164

[b64] Roth M. M., Helmchen F. & Kampa B. M. Distinct functional properties of primary and posteromedial visual area of mouse neocortex. J. Neurosci. 32, 9716–9726 (2012). 22787057 10.1523/JNEUROSCI.0110-12.2012 PMC6622284

[b65] Alitto H. J., Moore B.D.t., Rathbun D. L. & Usrey W. M. A comparison of visual responses in the lateral geniculate nucleus of alert and anaesthetized macaque monkeys. J. Physiol. 589, 87–99 (2011). 20603332 10.1113/jphysiol.2010.190538 PMC3039262

[b66] Mollon J. D. “Tho’ she kneel’d in that place where they grew…” The uses and origins of primate colour vision. J. Exp. Biol. 146, 21–38 (1989). 2689563 10.1242/jeb.146.1.21

[b67] Willmer E. N. Colour of small objects. Nature 153, 774–775 (1944).

[b68] Williams D. R., MacLeod D. I. A. & Hayhoe M. M. Punctate sensitivity of the blue-sensitive mechanism. Vision Res. 21, 1357–1375 (1981). 7314519 10.1016/0042-6989(81)90242-x

[b69] Humanski R. A. & Wilson H. R. Spatial frequency mechanisms with short-wavelength-sensitive cone inputs. Vision Res. 32, 549–560 (1992). 1604842 10.1016/0042-6989(92)90247-g

[b70] Johnson E. N., Van Hooser S. D. & Fitzpatrick D. The representation of S-cone signals in primary visual cortex. J. Neurosci. 30, 10337–10350 (2010). 20685977 10.1523/JNEUROSCI.1428-10.2010 PMC2933431

[b71] Neitz M. & Neitz J. The uncommon retina of the common house mouse. Trends Neurosci. 24, 248–250 (2001). 11311361 10.1016/s0166-2236(00)01773-2

[b72] Curcio C. A. *et al.* Distribution and morphology of human cone photoreceptors stained with anti-blue opsin. J. Comp. Neurol. 312, 610–624 (1991). 1722224 10.1002/cne.903120411

[b73] Petry H. M., Erichsen J. T. & Szel A. Immunocytochemical identification of photoreceptor populations in the tree shrew retina. Brain Res. 616, 344–350 (1993). 8358626 10.1016/0006-8993(93)90230-k

[b74] Prusky G. T., West P. W. R. & Douglas R. M. Behavioral assessment of visual acuity in mice and rats. Vision Res. 40, 2201–2209 (2000). 10878281 10.1016/s0042-6989(00)00081-x

[b75] Oyster C. W. The Human Eye: Structure and Function. (Sinauer Associates, Inc., 1999).

[b76] Hut R. A., Scheper A. & Daan S. Can the circadian system of a diurnal and a nocturnal rodent entrain to ultraviolet light? J. Comp. Physiol. A 186, 707–715 (2000). 11016786 10.1007/s003590000124

[b77] Tankersley C. G., Irizarry R., Flanders S. & Rabold R. Circadian rhythm variation in activity, body temperature, and heart rate between C3H/HeJ and C57BL/6J inbred strains. J. Appl. Physiol. 92, 870–877 (2002). 11796704 10.1152/japplphysiol.00904.2001

[b78] Chen T. W. *et al.* Ultrasensitive fluorescent proteins for imaging neuronal activity. Nature 499, 295–300 (2013). 23868258 10.1038/nature12354 PMC3777791

[b79] Rahim A. A. *et al.* In utero administration of Ad5 and AAV pseudotypes to the fetal brain leads to efficient, widespread and long-term gene expression. Gene Ther. 19, 936–946 (2012). 22071970 10.1038/gt.2011.157

[b80] Pennesi M. E., Lyubarsky A. L. & Pugh E. N. Jr. Extreme responsiveness of the pupil of the dark-adapted mouse to steady retinal illumination. Invest. Ophthalmol. Vis. Sci. 39, 2148–2156 (1998). 9761294

[b81] Jacobs G. H. & Williams G. A. Contributions of the mouse UV photopigment to the ERG and to vision. Documenta ophthalmologica. Adv. Ophthalmol. 115, 137–144 (2007). 10.1007/s10633-007-9055-z 17479214

[b82] Lyubarsky A. L., Daniele L. L. & Pugh E. N. Jr Recovery phase of the murine rod photoresponse reconstructed from electroretinographic recordings. J. Neurosci. 16, 563–571 (1996). 8551340 10.1523/JNEUROSCI.16-02-00563.1996 PMC6578659

[b83] Ji N., Milkie D. E. & Betzig E. Adaptive optics via pupil segmentation for high-resolution imaging in biological tissues. Nat. Methods 7, 141–147 (2010). 20037592 10.1038/nmeth.1411

[b84] Ko H. *et al.* The emergence of functional microcircuits in visual cortex. Nature 496, 96–100 (2013). 23552948 10.1038/nature12015 PMC4843961

[b85] Kerlin A. M., Andermann M. L., Berezovskii V. K. & Reid R. C. Broadly tuned response properties of diverse inhibitory neuron subtypes in mouse visual cortex. Neuron 67, 858–871 (2010). 20826316 10.1016/j.neuron.2010.08.002 PMC3327881

[b86] Li Y. *et al.* Clonally related visual cortical neurons show similar stimulus feature selectivity. Nature 486, 118–121 (2012). 22678292 10.1038/nature11110 PMC3375857

[b87] Carandini M. & Ferster D. Membrane potential and firing rate in cat primary visual cortex. J. Neurosci. 20, 470–484 (2000). 10627623 10.1523/JNEUROSCI.20-01-00470.2000 PMC6774139

[b88] Ringach D. L., Shapley R. M. & Hawken M. J. Orientation selectivity in macaque V1: diversity and laminar dependence. J. Neurosci. 22, 5639–5651 (2002). 12097515 10.1523/JNEUROSCI.22-13-05639.2002 PMC6758222

[b89] David S. V., Vinje W. E. & Gallant J. L. Natural stimulus statistics alter the receptive field structure of v1 neurons. J. Neurosci. 24, 6991–7006 (2004). 15295035 10.1523/JNEUROSCI.1422-04.2004 PMC6729594

[b90] Wang L., Sarnaik R., Rangarajan K., Liu X. & Cang J. Visual receptive field properties of neurons in the superficial superior colliculus of the mouse. J. Neurosci. 30, 16573–16584 (2010). 21147997 10.1523/JNEUROSCI.3305-10.2010 PMC3073584

