# Supplementary material for: Neuronal Representation of Ultraviolet Visual Stimuli in Mouse Primary Visual Cortex
Source: Sci Rep. 2015 Jul 29;5:12597. doi: 10.1038/srep12597 (PMC4518215; doi:10.1038/srep12597)
Supplement: Supplementary Information [file srep12597-s1.pdf]

Neuronal Representation of Ultraviolet Visual Stimuli in Mouse Primary Visual Cortex

Zhongchao Tan, Wenzhi Sun, Tsai-Wen Chen, Douglas Kim, Na Ji*

This PDF file includes:

Supplementary Figures 1

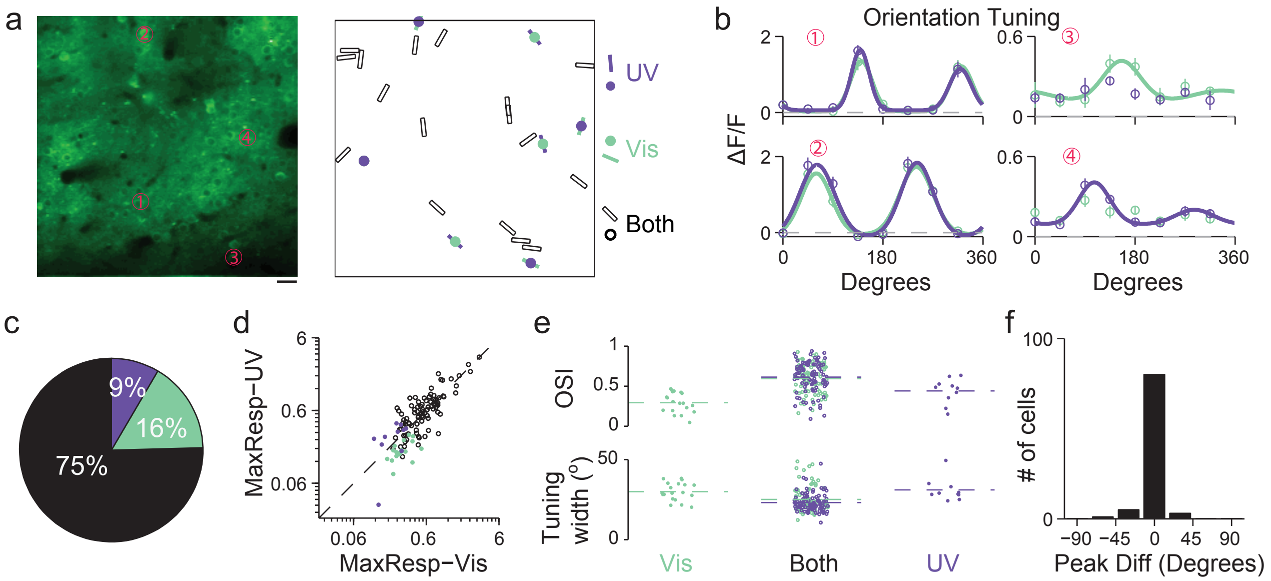

Supplementary Figure 1. Comparison of neuronal response in the primary visual cortex of Gnat$^{-/-}$ mouse to UV and visible stimuli. **(a)**, Left, example two-photon image of layer 2/3 neurons labeled with GCaMP6s. Scale bar: 20 μm. Right, responses to visible and UV drifting gratings. Dots, neurons with significant responses to visual stimulation. Bars, neurons with significant orientation selectivity. Purple, UV-only responsive; green, visible-only responsive; black, responsive to both wavelengths. **(b)**, Orientation tuning curves for four example neurons marked in **a**. Left, two neurons orientation-tuned for both visible and UV wavelengths. Right, two neurons exhibiting orientation selectivity to only visible and only UV drifting gratings, respectively. Open circles, mean; vertical lines, error bar, SEM.; curves, double Gaussian fits to mean data. Green: visible; purple: UV. **(c)**, Pie chart showing the percentage of neurons classified as orientation selective to only visible stimulation (green), only UV stimulation (purple), or both (black) (118 neurons in 3 animals). **(d)**, Scatter plot of the maximal response to UV versus that to visible gratings for neurons orientation-selective to only visible (green dot), only UV (purple dot), and both visible-and-UV (open black circle) gratings. **(e)**, Scatter plots of orientation selectivity index and tuning width. Dashed lines indicate mean values. **(f)**, Histogram distribution of difference in the preferred orientations of neurons that were orientation-selective to both UV and visible drifting-grating stimuli.
